# Effect of Peer-Based Low Back Pain Information and Reassurance at the Workplace on Sick Leave: A Cluster Randomized Trial

**DOI:** 10.1007/s10926-013-9451-z

**Published:** 2013-05-09

**Authors:** Magnus Odeen, Camilla Ihlebæk, Aage Indahl, Marjon E. A. Wormgoor, Stein A. Lie, Hege R. Eriksen

**Affiliations:** 1 Uni Health, Uni Research, P.O. Box 7810, 5020 Bergen, Norway; 2 Clinic Physical Medicine and Rehabilitation, Vestfold Hospital Trust, Stavern, Norway; 3 Section of Public Health, ILP, Norwegian University of Life Sciences, Ås, Norway; 4 Department of Health Promotion and Development, Faculty of Psychology, University of Bergen, Bergen, Norway

**Keywords:** Sick leave, Low back pain, Randomized controlled trial, Norway

## Abstract

*Purpose* To evaluate whether information and reassurance about low back pain (LBP) given to employees at the workplace could reduce sick leave. *Methods* A Cluster randomized controlled trial with 135 work units of about 3,500 public sector employees in two Norwegian municipalities, randomized into two intervention groups; Education and peer support (EPS) (n = 45 units), education and “peer support and access to an outpatient clinic” (EPSOC) (n = 48 units), and a control group (n = 42 units). Both interventions consisted of educational meetings based on a “non-injury model” and a “peer adviser” appointed by colleagues. Employees in the EPSOC group had access to an outpatient clinic for medical examination and further education. The control group received no intervention. The main outcome was sick leave based on municipal records. Secondary outcomes were self-reported pain, pain related fear of movement, coping, and beliefs about LBP from survey data of 1,746 employees (response rate about 50 %). *Results* EPS reduced sick leave by 7 % and EPSOC reduced sick leave by 4 % during the intervention year, while sick leave in the control group was increased by 7 % during the same period. Overall, Rate Ratios (RR) were statistically significant for EPSOC (RR = .84 (C.I = 0.71–.99) but not EPS (RR = .92 (C.I = 0.78–1.09)) in a mixed Poisson regression analysis. Faulty beliefs about LBP were reduced in both intervention groups. *Conclusions* Educational meetings, combined with peer support and access to an outpatient clinic, were effective in reducing sick leave in public sector employees.

## Introduction

Musculoskeletal pain is one of the most common causes of long-term sick leave and disability pension in Norway [1] and other European countries [2]. Low back pain (LBP) is the most common single diagnosis. In 2008, musculoskeletal pain was reported as the cause of 35 % of sick leave payments, and almost one in three new disability pensions in 2006 was granted for musculoskeletal disorders [3]. Employees in the public sector, especially females working in the municipalities has some of the highest sick leave levels in Norway [1]. The sick leave rate in the first quarter of 2011 was 8.1 % for the municipal sectors as compared to 6.1 % for the workforce as a whole.

While acute low back pain is usually short lasting [4], the prevalence of complaints and the rate of recurrence is high, the 1 year rate of recurrence reported to be between 24 and 80 % [5]. Development of maladaptive perceptions about the cause and prognosis of the low back pain is associated with a poorer clinical outcome [6]. Thus the prevention of the consequences of low back pain can also be seen as a way to improve the long term work participation for those with recurring low back pain, as well as decreasing the risk of low back pain becoming chronic.

A multitude of treatments have been developed for the prevention of LBP but the results have been disappointing [7]. It seems difficult to prevent acute non-specific LBP, but the consequences of LBP, such as fear of injury or movement, inactivity and/or sick leave can be prevented [7]. There is moderate evidence of the general effects of workplace interventions on sick leave [8]. Education and work adjustment to reduce LBP related sick leave, have shown mixed results [8–11], while interventions where employees, health practitioners, and employers are working together to implement work modifications are more consistently effective than other interventions [9, 11].

Brief Interventions (BI), based on the “non-injury model” proposed by Indahl [12, 13], has been among the most successful approaches to increase return to work for those with LBP [14–18]. According to this model, the back is considered a strong and robust structure, and pain is not to be taken as a sign of injury caused by any wrongdoing or ‘inappropriate’ behavior [19]. The non-injury model is different from the fear-avoidance model [20] in the understanding of why individuals choose activity or inactivity, and in that it is non-directive. Inactivity is a rational choice given an illness perception [21] that the back pain is caused by an injury, and likely to deteriorate with activity. This illness perception is replaced by a perception of LBP as a painful, but benign and usually self-limiting condition. The treatment providers’ job is not to “cure” the pain, nor to remove fear of movement, but simply to present the evidence for the benefit of being active [22] and let the employee decide. This non-injury model is consistent with the understanding and recommendations in the European Guidelines for the prevention of LBP [7].

Based on the BI and “non-injury model” a tailored work place intervention, “atWork”, has been developed. “atWork” is a continuation of the “Active back” intervention that showed a significant reduction in sick leave in a non-randomized trial [23]. Since the “Active back” trial, the intervention has been developed and standardized, and access to an outpatient clinic was added as an intervention component.

A main goal of atWork is to enable the individual to cope with acute LBP at the workplace. This is done through systematic education of all employees, and peer support/advice. The non-injury model and atWork has also a theoretical foundation from the Cognitive activation theory of stress (CATS) [24], where coping is defined as a positive response outcome expectancy, a belief that your actions or strategies will lead to a positive result. Changing expectations about LBP and sick leave, and creating positive response outcome expectancies [24] for being active and being at work despite pain, is the core of the intervention.

The aim of this study was to investigate if atWork, containing education and peer support with or without access to an outpatient clinic for severe and disabling pain had an effect on sick leave and beliefs about LBP.

## Methods

### Sample

All employees above 18 years of age, working at any of 135 units in the municipalities of Kongsberg and Horten, Norway, were invited to participate in the study. These municipalities are two medium sized cities with a population of about 25,000 each, located in the semi-urban part of south-eastern Norway. The number of municipal employees at the start of the intervention was estimated to be approximately 1,500 in Kongsberg and 2000 in Horten.

The 135 units were constructed, based on the municipalities “natural working units”, and would typically consist of employees who met regularly, and shared job tasks and/or physical location, such as employees working at the same school, nurses at a nursing home etc. A total of 5 different sectors were represented (see Table 1). Some privately run nursery schools collaborating closely with the municipality also participated with individual data, but the units did not provide sick leave data. In addition to register data on sick leave for all units, data on different health related parameters was collected from 1,746 individual employees (mean age 44.2 years (SD = 11.5), 81.5 % (1,422) women, mean years of education 14.5 years (SD = 3), and 22.3 % (387) managers. Of these, 860 (49.3 %) also responded to the follow up questionnaire 12 months later. There were no significant differences in age, gender, or education between those who responded to the follow up and those who did not.

**Table 1:** Demographic data for the two municipalities, based on questionnaire data

	Kongsberg	Horten	Total
N of questionnaire responders	755	991	1,746
Percentage female (n)	82.1 % (620)	81.0 % (802)	81.5 % (1,422)
Mean age (SD)	44.6 (11.4)	43.8 (11.5)	44.2 (11.5)
Mean years education (SD)	14.4 (3.3)	14.6 (2.8)	14.5 (3.0)
Percentage managers (n)	21 % (158)	23.3 % (229)	22.3 % (387)
*Percentage of types of workplaces*:			
Nursing homes for the elderly	28,4 %	21.6 %	20.7 %
Assisted living quarters for people with cognitive impairments	23.2 %	19.6 %	23.8.5 %
Schools	30.9 %	15.1 %	22.6 %
Nursery schools	7.6 %	23.6 %	13.8 %
Staff and others	9.9 %	20.1 %	19.1 %

### Procedure

Sick leave was assessed on the unit level; and musculoskeletal pain, pain related fear, general health, coping, and belief in myths about LBP on the individual level. Cluster randomization was chosen so that the intervention should include all cluster members regardless of their exposure to the intervention, according to the intention to treat principle [25].

The project started in Kongsberg in 2008 and in Horten in 2009. Information about the project was provided through a series of meetings with managers at all levels in the municipalities. The managers provided all employees with information about the study, including an information flyer, with information about the different interventions. By employing two local employees at the municipalities working for the project part time, more information about the project could be given on a one-to-one basis. Informed consent forms and questionnaires were distributed at the workplace, and the participating respondents signed informed consent forms and returned these together with questionnaires to Uni Health in sealed envelopes. Follow up questionnaires were administered 12 months later by mail and electronically, as well as through the workplace. As in any organization, the Human resources departments also ran other general efforts to reduce sick leave that was offered to all employees independent of the atWork project.

**Fig. 1:**
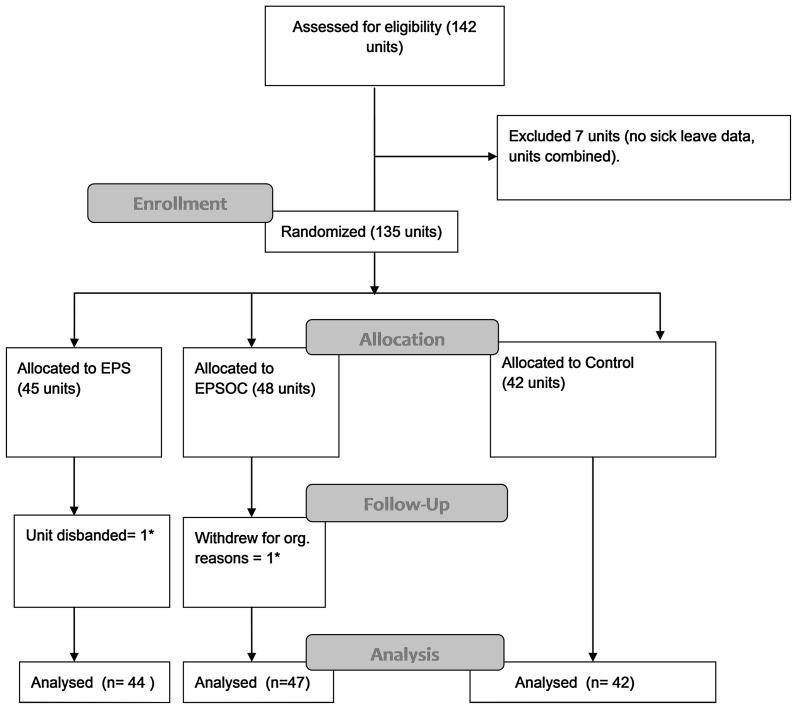
Flow chart of participants: *EPS* Education and Peer Support. *EPSOC* Education, Peer Support and Outpatient Clinic. *Included in poisson analysis according to intention to treat principle

Staff at the clinic for physical medicine and rehabilitation at the Vestfold hospital trust did recruitment of the municipalities and units. The 135 units of employees were randomized into three groups: (1) education and peer support (EPS) (n = 45 units), (2) education and peer support with access to an outpatient clinic (EPSOC) (n = 48 units), and (3) a control group (CON) (n = 42 units) (see Fig. 1). Concealed, stratified, randomization of whole units was done by the first author using computer generated, random numbers, at Uni Health. The units were stratified according to sectors only; i.e. schools, nursing homes, preschools. No leaders or employees at the municipalities, or any of the other members of the research or intervention team, could predict the outcome of the randomization/allocation before it was done. After the randomization, information about the allocation was given to the managers in all units. It was not possible to blind the participants of their allocation, due to the nature of the intervention. The main outcome variable of the study was obtained by register data of sick leave, and thus blinded.

### Primary Outcome Measure: Sick Leave (Unit Level)

Sick leave was measured with data from the records of the municipalities. The municipalities kept central records over the total sum of agreed work days for all employees in each unit, and how many of these days were lost to sick leave in total (both self-certified and physician certified). Agreed work days are the number of days that the employee is expected to come to work according to the employment contract. Days lost to sick leave included sick leave for any diagnosis, and the data were from all the employees of the randomized units and not only those responding to questionnaires. Sick leave data was not reported for individual employees, only units. The municipalities counted sick leave by *positions* and not *persons employed* in each unit (so called “head count”), therefore the precise number of individuals contributing to the summed data was not available, only the number of full time positions. The number of allocated positions was also likely to change across the intervention, but this did not affect the ratio of agreed to lost work days. Sick leave data was collected for the year before the intervention, and the year of the intervention, measured from the start of the intervention.

One unit chose to leave the intervention for organizational reasons, but data from this unit was included for intention to treat analysis. 5 units did not report sick leave data to the central register of the municipality, but kept their own records, which were inaccessible for us. These were excluded from the sick leave analysis, but the employees were allowed to contribute survey data. Two units lacked follow up data because they were disbanded during the intervention period.

### Secondary Outcome Measures, Individual Level

*Musculoskeletal complaints* were measured by a subscale of The Subjective Health Complaint Inventory (SHC) [26], a reliable and valid measure of common health complaints [26, 27] which consists of 29 questions concerning subjective somatic and psychological complaints experienced during the last 30 days. The severity of each complaint is rated on a 4-point scale (0 ~ none, 1 ~ some, 2 ~ much, 3 ~ severe). The musculoskeletal factor consists of eight items: headache, neck pain, upper back pain, low back pain, arm pain, shoulder pain, migraine and leg pain during physical activity.

*Low back pain* was measured by a single item from SHC, asking if you have had low back pain during the last 30 days, rated on a 4-point scale (0 ~ none, 1 ~ some, 2 ~ much, 3 ~ severe).

*General health* was measured with a single item; “On the whole, how would you say that your health is?” scored on a five-point scale ranging from “very bad” to “very good”. The bad and very bad categories were combined so that the scale had four categories from bad/very bad (1) to very good (4). Single item global health measures have been extensively validated as a measure of health [28].

*Attitudes and beliefs to LBP* were measured by two questions taken from Deyo’s “back pain myths” [2, 29]. The myths represent untrue and maladaptive beliefs about low back pain [29]. Two of the most prevalent myths “Most back pain is caused by injury and heavy lifting” and “Everyone with back pain should have a spine X-ray” were used, since these were specifically addressed in the intervention. The myths were scored on a five point Likert-scale from “completely disagree” to “completely agree”. These were classified as beliefs in myth (4 and 5) and non-beliefs in myth (1–3).

*Coping was measured by The Theoretically Originated Measure of the Cognitive Activation Theory of Stress (Tomcats),* a new inventory developed to measure the concept of response outcome expectancies in the CATS theory [24]. The inventory has been tested in the general Swedish population [30]. The inventory consisted of 6 statements about response outcome expectancies representing the three response outcome expectancy variables of CATS; coping (one item), helplessness (two items) and hopelessness (three items). The questions were recoded from a five to a four point scale in order to make it comparable with other studies. After recoding, all items were ranged on a four point scale from “not true at all” (1) to “completely true”(4).

*Pain related fear was measured by The Tampa scale of kinesiophobia* [31], a reliable and valid measure of pain related fear in acute low back pain [32]. The scale has 13 items with a 4 point scale from strongly disagree (1) to strongly agree (4). A mean value of all items was calculated.

### Interventions

#### Education and Peer Support Group (EPS)

Two educational meetings, with approximately 2–3 months between them, were offered to all employees in the EPS and EPSOC groups. Each of the educational meetings lasted for 45 min, and the purpose was to educate the employees and leaders about LBP. Myths of back pain: such as the consequence of inactivity and bed rest, or the value of imaging like X-rays and MRs, were discussed with the employees. The main message was the non-injury model and the evidence for it. It was discussed how the working environment could be well suited for working, even if a person had LBP, and how the employees felt about going to work if they had LBP. Presenters, mostly physiotherapists, who had undergone extensive training, led the meetings.

During the first Educational Meeting, “Peer advisers” for each of the units were recruited among the employees, either by volunteering or agreeing after being suggested by fellow workers. The Peer adviser was a fellow worker, with no former training in medicine or related fields. All Peer advisers were given information and training at one of the outpatient clinics for two days, and could contact the outpatient clinic at any time for general help or for help with specific cases. The training focused on more in depth knowledge of the back, LBP, and instruction on how to be a Peer adviser. The Peer advisers were also given a booklet with more information, and a book explaining LBP in layman’s terms [33].

When necessary, the Peer advisers offered help with work modification, in order to increase the employees’ likelihood to stay at work. If an employee had persistent LBP, or felt unsure about the nature of the back pain, the Peer advisers were instructed to advise them to contact their GP, or if assigned to EPSOC, to contact the outpatient clinic. The Peer adviser’s role was not to give a diagnosis or to recommend treatment options, but to give social support, and to use their knowledge of the working environment to help their colleagues with staying at work, despite having pain.

#### Education, Peer Support and Occupational Clinic Group (EPSOC)

In addition to the Educational Meetings and access to a Peer adviser, EPSOC included access to medical evaluation and treatment, at one of two outpatient clinics. When an employee experienced LBP, the Peer adviser could refer the employee directly to the clinic if required and/or requested. All employees referred to the clinic, went through an initial individual assessment, including a physical examination by a physiotherapist, followed by two reinforcing educational workshops with other patients, where the message of the Educational Meetings was repeated. The examination was done in order to screen for any condition requiring further medical assessment or treatment, and to give the employee insight and reassurance. The goal was to make the employee feel confident about the robustness of their spine and consequential confidence about the benefit of staying active, including going to work. In case additional medical care or assessment was required, the employee was referred to relevant specialist care.

The interventions in the EPS and EPSOC groups were not targeted to those who had previously experienced LBP. A medical doctor and specialist in rehabilitation medicine had the overall medical responsibility. Medical doctors were also available to the outpatient clinics in cases requiring medical help.

#### Control Group

The control group, as well as the intervention groups, continued to receive usual care from their general practitioners and the Norwegian health care system. However, the control group did not receive any intervention from the project in addition to this.

### Ethics

The study followed the Helsinki declaration. The project was approved by the Norwegian regional ethics committee in western Norway (REK-vest, ID 6.2008.117), the Norwegian social science data services recommended the study (NSD, ID 18997), as well as the privacy ombudsman at the National Hospital (Rikshospitalet, ID 08/2421). Participation in the project was voluntary, and participants could withdraw consent at any time in the project.

There were no restrictions on the employees to seek any other treatments during the intervention, or on other preventive strategies run by the municipalities.

### Statistics

#### Power Calculation

For the main outcome (sick leave), we used all the units available in the two municipalities. Recruiting more municipalities had to be abandoned due to practical reasons. The power calculation for our analysis was based on 1,000 bootstrap replicates of the observed number of units and sick leave for these units. From the simulations we approximated that we had a power of .82 for a 25 % relative change in sick leave, and a power of .30 for a 10 % change, with the sample size of 39 units per group.

For the continuous secondary outcomes, we calculated that a sample size of 432 would be sufficient to detect an effect size of .15 in the sample with a power of .80. The smallest sample size in the final dataset was 621. For the dichotomous secondary outcomes, a sample size of 268 would give a .80 power to detect a difference in proportions of .15. The smallest sample size in the data was 458.

### Data Analysis

A Generalized Linear Mixed effects Poisson Model (GLMM), as given in formula (1), was used to investigate possible effects of the interventions on sick leave. The control group was set as the reference to which the two interventions were compared.

1 $$ \log (D_{ij} ) = {\mathbf{x}}_{ij}^{T} \beta + {\mathbf{z}}_{ij}^{T} u_{i} + \log (N_{ij} ) $$

This approach was chosen to account for the random variation in sick leave days (u, assumed to be normally distributed) between the 135 units (i) measured repeatedly (j) over time. The z matrix is a design-matrix to adjust for the random variation between units over time and to adjust for over-dispersion (variation between the units that exceeds the theoretical Poisson variation), while x is the design-matrix where comparisons of the groups are done at follow-up, but not at baseline [38].

The rationale for using the GLMM, with a Poisson distribution, was to maintain the separate measures for each of the units, for the two time points, instead of aggregating the data before the analyses. The relative size of the units (in agreed work days) was thus preserved in the analysis. Both the baseline and the follow-up measures were included as outcomes in the model, while differences between the intervention groups and the control group were estimated for the follow-up only. This is equivalent to test the changes between the intervention groups from baseline to follow up. The outcome from this regression model is rate ratios (RRs). The GLMM analyses were performed in the lme4 library [34] in the statistical program R, version 2.11.1 [35].

For the continuous secondary measures, we first subtracted the baseline scores from the follow up scores to obtain the change in scores. Then the change was tested in an ANOVA analysis to test if there were significant group differences. For the dichotomous outcomes, we first calculated those improved, unchanged and worsened from baseline to follow up. A McNemar test was used to test if the change was significant. Pairwise group differences between intervention and control in these proportions were then tested for statistical significance with a chi–square test. SPSS statistics version 18.0. (Chicago: SPSS Inc) was used for these analyses.

## Results

### Primary Outcome Measure—Sick Leave

Sick leave was reduced by 7 and 4 % for EPS and EPSOC groups respectively, and increased by 7 % in the control group (Table 2).

**Table 2:** Sums and percentages of work days lost due to sick leave in the two municipalities and totally, divided by randomization group (N of units, baseline = 135, 1 year = 133)

		Baseline			1 year			Change	
	N	Days agreed	Days lost	Sick leave (%)	Days agreed	Days lost	SICK leave (%)	Percentage	Percent points. (%)
*Kongsberg*									
EPS	16 (15)	100946	11023.1	10.92	89435	7871.8	8.80	−19.41	−2.12
EPSOC	19	122979	11004.4	8.95	130949	11758.8	8.98	0.34	0.03
Control	14	68524	5709.1	8.33	67141	6562.9	9.77	17.29	1.44
*Horten*									
EPS	29	124718	14865.7	11.92	132574	15872.1	11.97	0.42	0.05
EPSOC	29 (28)	111641	12402.6	11.11	108258	11165.3	10.31	−7.20	−0.80
Control	28	111125	11974	10.78	108892	12026.4	11.04	2.41	0.26
*Total*									
EPS	45 (44)	225664	25888.8	11.47	222009	23743.9	10.70	−6.71	−0.77
EPSOC	48 (47)	234620	23407	9.98	239207	22924.1	9.58	−4.01	−0.40
Control	42	179649	17683.1	9.84	176033	18589.3	10.56	7.32	0.72

*EPS* education and peer support, *EPSOC* education, peer support and outpatient clinic

**Fig. 2:**
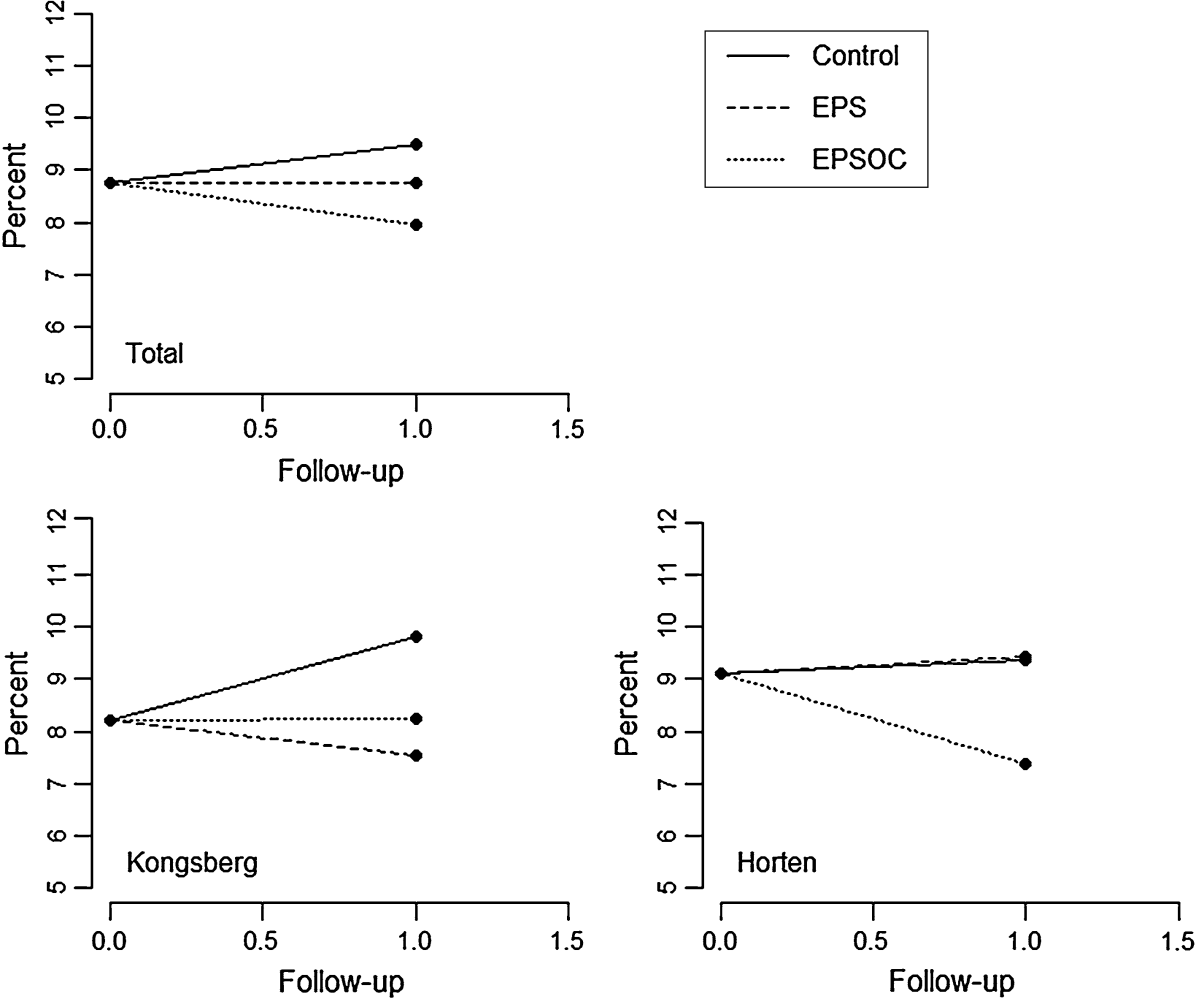
Mixed Poisson regression model adjusted of the effect of the intervention on change in sick leave for both municipalities and the whole sample. *X axis* represents time in years and the *Y axis* represents percentage sick leave

Compared to the control group, there was an overall significant reduction in sick leave in the EPSOC group (p = 0.04). The effects were not significant in the individual municipalities, however, although there was a trend towards significance in both Kongsberg (p = 0.07) and Horten (p = 0.08). In the municipality of Kongsberg, sick leave was significantly reduced in the EPS group (p = 0.004) but there was no effect of EPS in Horten (see Table 3 and Fig. 2).

**Table 3:** Mixed Poisson regression of unit sick leave change from the year before the start of the intervention to the intervention year

	Rate	CI	*p* value
	Ratio		
*Kongsberg (n of groups* *=* *49)*			
EPS	0.77	(0.64 to 0.92)	0.004
EPSOC	0.84	(0.70 to 1.01)	0.07
Control	1		
*Horten (n of groups* *=* *86)*			
EPS	1.01	(0.80–1.27)	0.96
EPSOC	0.79	(0.60–1.03)	0.08
Control	1		
*Total (N of groups* *=* *135)*			
EPS	0.92	(0.78–1.09)	0.35
EPSOC	0.84	(0.71–0.99)	0.04
Control	1		

The model controls for initial differences in sick leave and retains the relative size of the units. Control is the comparison group

*CI* 95 % Confidence interval, *EPS* education and peer support, *EPSOC* education, peer support and outpatient clinic

### Secondary Outcomes

#### Low Back Pain and Musculoskeletal Pain

In the EPS group, 155 (54 %) of the employees reported LBP before the intervention, and 159 (56 %) after the intervention year. For the employees in the EPSOC group there was a significant decrease in number of employees reporting LBP during the intervention year from 159 (58 %) to 136 (49 %) (χ^2^ = 5,97, p = 0.015). The control group had an increase from 105 (54 %) to 112 (58 %). Musculoskeletal complaints were reported by (84–89 %) of the respondents in all groups, and no significant changes were detected in any group.

#### Belief in Low Back Pain Myths

At baseline, there were 109 employees in the EPS group (38 %), 86 in the EPSOC group (32 %) and 66 of the control group (34 %) who believed that lifting was usually the cause of back pain. After the intervention year, believers of the myth was reduced to 38 employees in the EPS group (reduction of 65 %), 41 in the EPSOC group (reduction of 52 %) and to 58 in the control group (reduction of 12 %). For the belief that “everyone with LBP should have an x-ray”, the reduction was more equal in the groups, with EPS reduced from 71 (25 %) to 30 (11 %), EPSOC from 73 (27 %) to 39 (14 %) and the control group from 48 (26 %) to 39 (21 %). The reductions were 58, 47 and 19 % respectively.

There was a significant reduction in pain related fear in all groups as well as a small, but significant increase in helplessness in the EPS group and in general health in the control group (see Table 4). The mean reduction in belief in the two back pain myths was significant in both intervention groups as compared to the control group, but no significant group differences in the change in any of the other outcomes.

**Table 4:** Level of pain related fear, general health, coping, helplessness, and hopelessness and belief in low back pain myths for all intervention groups, at baseline and one year, mean (SD)

	EPS					EPSOC			Control			
	Baseline		1 year			Baseline		1 year	Baseline		1 year	
	N	Mean (SD)	N	Mean (SD)	N	Mean (SD)	N	Mean (SD)	N	Mean (SD)	N	Mean (SD)
Pain-related fear	570	1.96 (.46)	304	1.74 (.55)^2^	570	1.92 (.48)	360	1.72 (.47)^2^	379	1.93 (.46)	214	1.82 (.53)^2^
General health	566	3.00 (.79)	359	3.07 (.83)	572	3.03 (.78)	399	3.06 (.75)	378	3.05 (.78)	253	2.98 (.84)^a^
Coping	573	3.36 (.44)	360	3.36 (.41)	580	3.36 (.41)	403	3.36 (.39)	385	3.37 (.41)	261	3.39 (.41)
Helplessness	574	1.56 (.58)	362	1.61 (.58)^a^	581	1.58 (.63)	404	1.56 (.58)	384	1.51 (.56)	260	1.53 (.59)
Hopelessness	572	1.49 (.48)	361	1.50 (.48)	579	1.49 (.49)	404	1.47 (.49)	384	1.49 (.48)	260	1.47 (.46)
Myth-lifting^4^	562	3.31 (.90)	359	2.70 (.95)^b,c^	560	3.18 (.85)	398	2.84 (.92)^b,c^	377	3.23 (.94)	257	3.19 (.92)
Myth-X-ray^e^	552	3.02 (1.11)	358	2.34 (1.05)^b,c^	554	2.94 (1.04)	397	2.44 (1.07)^b,c^	375	2.93 (1.13)	253	2.87 (1.01)

^a^Difference is significant at the 0.05 level

^b^Difference is significant at the 0.001 level

^c^Significantly different from the control group in an ANOVA analysis with a Scheffe test

^d^Most back pain is caused by injuries or heavy lifting

^e^Everyone with back pain should have a spine X-ray

### Satisfaction with the Intervention

In the intervention groups, 63.5 % of the respondents participated in at least one information meeting, and 51.7 % participated in both information meetings. Of those who reported that they had attended information meetings (n = 697), 72 % indicated that the information given was trustworthy “to some degree, large degree or very large degree”. Of those who reported that they had seen the peer advisor (n = 64), 62 % indicated that the information was trustworthy. Of those survey respondents who had used the outpatient clinic (n = 24) 85 % indicated that the information was trustworthy.

Due to the low response rate, a phone survey of 60 Peer advisers was done to estimate the usage of the peer advisors. The survey showed that 14 employees had been in contact from the EPS group and 64 from the EPSOC group. Twenty-six employees from Kongsberg and 19 from Horten were referred to the outpatient clinic during the intervention period. Those in need of further medical attention were referred to specialist care in cooperation with the patients GP, but this was very rarely necessary.

The intervention was generally well received by employers and employees. Participation in the project was voluntary, so it is likely that those negative to the intervention chose not to participate. There were no adverse events reported in the study.

## Discussion

Overall, there was a significant effect on sick leave, for the Education, Peer Support and Occupational clinic group (EPSOC) intervention compared with the control group. The Education and Peer support (EPS) showed a strong effect in Kongsberg, but was not significant overall. Although there was only a trend towards significance in both municipalities, EPSOC reduced sick leave more uniformly in units across both municipalities, leading to a stronger effect in the mixed model GLMM.

While the sample was very large, it is not likely that the analysis were sufficiently powered to detect the relatively small changes in sick leave. The education and Peer Support (EPS) intervention gave a similar sick leave reduction to that of the EPSOC group, but this difference was not significant overall, and a lack of power may be part of the reason for the differences between the interventions.

A replication of the intervention is the only way to determine if the lack of an effect of the EPS intervention was due to the lack of the outpatient clinic component, or caused by other external factors. For instance, Horten municipality participated in the intervention while a downsizing was underway, which may have affected the EPS group more than the EPSOC group, due to the former being slightly more workplace based.

In general, there was a consistent pattern that the intervention groups reduced their sick leave compared to the control group, with the exception of the EPS group in Horten that had no effect. While the effect was modest, the large number of employees in the municipalities means that it is likely to have a considerable impact for the economy and quality of services provided by the municipalities, especially if it is possible to sustain the effect over time.

While the RCT design gives a general protection against external effects (so called “third-variables”) it is possible that there were differences in how such variables affects the units in the different randomized groups. The units were stratified by unit type, but the uneven size of the units indicates that such external factors cannot be completely excluded.

The intervention was done with two different outpatient clinics. The clinics agreed on a common protocol for their intervention, and observations were done to ensure adherence to the protocol. The overall impression was that the message delivered, and the way it was delivered was identical in both municipalities.

Data on compliance suggested that any differences between EPS and EPSOC were not due to utilization of the outpatient clinic, which received few employees for treatment in both municipalities. There was also no indication that the Peer adviser was frequently used. This indicates that the difference between the groups is more likely to be caused by how the intervention was perceived by the employees rather than how much the intervention was used. The outpatient clinic may have functioned as an “insurance” against potential pain from activity, which may have made the employees feel more confident in going to work, knowing that they would get fast access to treatment in case they needed it.

The effects on sick leave are interesting since there have been a number of randomized trials reporting limited or no effects on sick leave from educational worksite programs [10, 36], integrated health programs [37, 38], workplace screening, and/or ergonomic interventions [39–41]. An educational pamphlet on advice and reasons to stay active showed promising results [42], and a non-randomized study of the precursor to the atWork intervention also had a significant effect on sick leave [23], as did an integrated care approach [43].

The intervention was done as a pragmatic RCT, within a very realistic context. Even though the peer advisor and outpatient clinics were infrequently used, the intervention significantly reduced sick leave in both municipalities. The effects seem independent on the rate of utilization and are likely to be similar to the results that could be expected in a real life setting. The absolute sick leave reductions were similar to those achieved in the active back trial [23], which further supports the validity of the findings.

### Secondary Outcomes

The EPSOC group had a significant reduction in Low Back Pain (LBP) but this was not significantly different from the control group. Also, musculoskeletal pain in general was not reduced. This is in line with previous findings [15, 44].

Pain related fear showed a small, but statistically significant reduction in all groups. Although there was a significant increase in helplessness in the EPSOC group, the increase was too small to indicate any meaningful change in helplessness, as was the very small significant decrease in general health in the control group.

The message at the educational meetings was aimed at giving the evidence against Deyo’s myths [29]. The belief in two of the myths was reduced in both intervention groups, indicating that the message had been understood and accepted. This is important in light of a non-injury model approach, where changing maladaptive ideas are important [13].

## Limitations and Implications

The municipalities’ sick leave registers were used in the analysis, making it possible to do a full intention to treat analysis. One challenge is that the initial sick leave levels varied between the groups, mainly due to variation in size and sick leave levels. We controlled for this variation by using the mixed Poisson regression analysis where the baseline data was fixed.

The sample in the atWork study is unique in its diversity compared to other workplace studies comparing employees with similar work tasks and environment. This made the sample much less vulnerable to effects of location or group specific effects. However, this might also be some of the reason why the response rates on the individual data were low, estimated at around 50 % at baseline. In addition to this there was also a somewhat large loss to follow up from baseline. Some of this may have been due to normal turnover of employees that occur in most workplaces. Together, the low response rate and loss to follow up may have introduced selection bias to the secondary outcomes. For the secondary outcomes, the results were not controlled for possible confounders, and a multilevel design was not used. This was primarily done to preserve statistical power for the analysis, and since the RCT design was considered sufficient to ensure equal groups. However, due to the fact that the study was cluster randomized, possible group effects or confounding effects on the results cannot be completely ruled out.

The major strengths of the study were the large size, full data set on sick leave, and the applied setting. The major limitations were the lack of individual sick leave data, and the low response rate and loss to follow up in the survey data in the secondary outcomes.

## Conclusions

The primary purpose of atWork was to prevent negative consequences of LBP, such as sick leave. There was a significant effect of EPSOC on sick leave when compared to the control group. EPS had mixed results, and no firm conclusion can be drawn on its effectiveness. Both interventions also decreased faulty beliefs about low back pain.

The combination of feasibility of the intervention, and its promising effects on sick leave, gives good reasons to consider the non-injury model as a viable alternative approach to traditional worksite interventions.
